# Dynamic changes in the appropriateness of urinary catheter use among hospitalized older patients in the emergency department

**DOI:** 10.1371/journal.pone.0193905

**Published:** 2018-03-22

**Authors:** Fang-Wen Hu, Hsin-I Shih, Hsiang-Chin Hsu, Ching-Huey Chen, Chia-Ming Chang

**Affiliations:** 1 Department of Nursing, National Cheng Kung University Hospital, Tainan City, Taiwan; 2 Department of Emergency Medicine, National Cheng Kung University Hospital, Tainan City, Taiwan; 3 Department of Medicine, College of Medicine, National Cheng Kung University, Tainan City, Taiwan; 4 Department of Nursing, College of Health Sciences, Chang Jung Christian University, Tainan City, Taiwan; 5 Division of Geriatrics and Gerontology, Department of Internal Medicine, National Cheng Kung University Hospital, Tainan City, Taiwan; University of Alberta, CANADA

## Abstract

**Objectives:**

To investigate incidence, rationales, related factors and outcomes for changing from appropriate catheter placement to inappropriate use among hospitalized older patients in the emergency department.

**Methods:**

A secondary analysis was adopted from a longitudinal study that was designed to follow the lifecycle of the urinary catheter among hospitalized older patients. Patients aged 65 and older with a urinary catheter that had been placed in the emergency department were included. Demographic factors, present health conditions, conditional factors of catheter placement, and rationales for daily urinary catheter use were collected from the original data. Inappropriate urinary catheter days were evaluated as an outcome.

**Results:**

Appropriate urinary catheters were placed in the emergency department in 117 of the 156 patients (75%). Of these patients, 77 patients (65.8%) experienced a change from appropriate placement to inappropriate use, with a mean duration of 2.88±1.56 days. The common rationales were post-operation for hip fracture (36.3%) and no longer needing to monitor urine output (27.2%). A hierarchical regression model shows that a change from appropriate catheter placement to inappropriate use was associated with a diagnosis of urinary tract infection (OR = 0.15; 95% CI = 0.03–0.77; *p* = 0.02) and no record of the indication for catheter placement (OR = 4.76; 95% CI = 1.20–18.90; *p* = 0.02), and all variables together explained 35.9% of the variance. In addition, a change from appropriate placement to inappropriate use was further associated with prolonging inappropriate catheter-days (β = 5.34; 95% CI: 3.72–6.97; *p* <0.001).

**Conclusions:**

The study highlights a considerable percentage of change from appropriate placement to inappropriate use. Efforts to construct reminder intervention, to improve the record of catheter placement and continued attention to catheter use are necessary to reduce inappropriate urinary catheter use.

## Introduction

### Background and importance

Catheter-associated urinary tract infection (CAUTI) is one of the most common health care-associated infections (HAIs), accounts for 34% of all HAIs [1] and is associated with significant morbidity and excess health care costs [2]. Hospitalized older patients are disproportionately affected, as they more commonly receive urinary catheters and are more susceptible to associated complications [3]. In addition to CAUTI, this population is more vulnerable to the noninfectious complications of urinary catheter use, including falls, delirium, urethral trauma and pain [4, 5].

Appropriateness of urinary catheter use is defined as using a urinary catheter with evidence of a medical indication such as surgery, urinary retention or a condition warranting accurate measurement of urinary output [6]. Key concepts for reducing catheter-associated adverse outcomes are lists of appropriate and inappropriate catheter medical indications, which restrict use to appropriate indications and prompt catheter removal when catheters are no longer appropriate. In particular, it is more likely that a urinary catheter is placed in hospitalized older patients without a medical indication, and of these, up to 43.9–54% are inappropriately used [7–9]. Nearly half of hospitalized patients are admitted from the emergency department (ED), where decisions to place urinary catheters are often made. Therefore, recent studies have focused on the ED, with efforts aimed primarily to reduce the unnecessary placement of urinary catheters [10–13]. As an integral part of urinary catheter use, we must not only cautiously select appropriate patients for catheter placement in the ED but also re-evaluate appropriateness at transitions (shift change, hospital admission, floor transfer or discharge). The ED can be viewed as the “front door” or “point of entry”, where efforts to reduce unnecessary urinary catheter use should be directed.

### Goals of this investigation

From previous work, we have shown that inappropriate urinary catheter placement and inappropriate catheter-days were related to worsening clinical outcomes in older patients [14, 15]. There is also a significant body of literature aimed at minimizing urinary catheter placement in the ED and reducing the duration of each catheterization during hospitalization [16, 17]. However, there was insufficient evidence to support the effectiveness of the intervention. An understanding of the dynamic change in the appropriateness of urinary catheter use is crucial for further intervention. Therefore, this study investigated the incidence, rationales, related factors and outcomes for changing from appropriate to inappropriate catheter use among hospitalized older patients in the ED.

## Methods

### Study design and population

The current study is a secondary analysis of patients enrolled in a longitudinal study that was designed to follow the lifecycle of the urinary catheter among hospitalized older patients in an earlier study [14, 15]. Between October 1, 2012 and October 31, 2013, convenient sampling was used to recruit patients from all adult wards, except obstetrics and gynecology and hospice, of an 1135-bed tertiary-care medical center in southern Taiwan. Patients aged 65 and older with a urinary catheter placed within 24 hours of hospitalization were enrolled. Those who had a urinary catheter placed before admission or were transferred to intensive care units or hospice services were excluded. There were 327 patients who agreed to participate; six patients who were transferred to the intensive care units were excluded from the study, leaving 321 patients who provided data. To assemble a study sample of patients who had received a urinary catheter in the ED, 165 patients were excluded because they had urinary catheter placement in the wards or the operating room, leaving an analytic sample of 156 patients for this study.

The study was approved by the Institutional Review Board of the study hospital. Written informed consent was obtained; if a patient lacked the capacity to consent, a proxy’s consent was obtained.

### Methods and measurement

In the study hospital, the use of urinary catheters was based on physician judgment with no standard criteria deemed necessary. The lack of clinical guidelines for urinary catheter use required the development of appropriate indications for catheter use for the purpose of the study. Indications of appropriateness for the use of urinary catheters from the original study were developed by literature review [6, 8, 18–20] and validated through expert consensus. Five medical experts from the study hospital were consulted, among them two were from geriatrics, one from urology, rehabilitation medicine and infectious disease. The content validity indices (CVI) is 0.87 and urinary catheters were considered appropriate for any of the following indications: neurogenic bladder dysfunction (where intermittent catheterisation is not possible), urinary retention or bladder outlet obstruction, medication instillation or bladder irrigation, conditions that might warrant accurate measurement of urinary output in critically ill patients, altered mental status or unresponsive, patients undergoing urologic surgery or other surgery on the contiguous structure of the genitourinary tract, hip fracture, perioperative management in surgical patients (preoperative catheter insertion for patients going directly to the operating department, anticipated prolonged duration of surgery, anticipated need for large-volume infusions or diuretics during surgery, need for intraoperative monitoring of urinary output, immediate postoperative management in surgical patients), and open sacral or perineal wounds with a need for urinary diversion in incontinent patients. Other indications that were not listed in the indications of appropriateness were identified in this study as inappropriate use of urinary catheters. In addition, for postoperative management in surgical conditions, based on the Healthcare Infection Control Practices Advisory Committee (HICPAC) guideline, the presence of a urinary catheter in operative patients over 24 hours postoperatively was identified as inappropriate use, unless there were previously mentioned appropriate indications [6].

Data were collected from the original study with several strategies by two researchers, which included interview, medical records review and observation. Inter-rater reliability of medical records abstraction was assessed using duplicate review of medical records of 10% of patients. Besides, Inter-rater reliability of interview and observation was evaluated the same patient by two researchers of consecutive five patients. All inter-rater reliability of interview, medical records review and observation (Cronbach’s alpha) is 0.96.

After obtaining consent, data collection started within 48 hours of hospitalization. The medical records included demographic factors (gender and age), present health condition (Charlson Comorbidity Index and urinary tract infection diagnosis) and the presence of the rationale for urinary catheter placement (yes or no). The Charlson Comorbidity Index indicates the number and severity of comorbidities. Scores range from 0 to 37, with a higher score indicative of more severe comorbidities [21]. The medical records of physicians’ and nurses’ notes were reviewed to clarify indications for urinary catheter placement. If the medical record did not explicitly document the indication for catheterization, the patients’ physician or primary care nurse was consulted. If there was no reliable information for the indication, the inappropriate catheter use was coded as “no evident reason for use of urinary catheter”, and this condition was identified as inappropriate use. The interview was carried out with the Short Portable Mental Status Questionnaire (SPMSQ) [22] and the Geriatric Depression Scale Short-Form (GDS-SF) [23] for those who were able to communicate. Cognitive impairment was defined as 2 or more errors after adjusting for education level on the SPMSQ [24]. For patients who could not respond to the SPMSQ, cognitive impairment was automatically recorded. Depressive symptoms were defined as a total score of more than 8 on the GDS-SF [25]. Urinary incontinence prior to admission was also determined during the interview. If patients were incompetent in communication, their main caregiver was asked. Urinary incontinence was defined as patient reported urine leakage within the previous 2 weeks [26]. In addition, the visual analog 0–10 was used to measure caregivers’ perspective of convenience, patients’ perspective of convenience and comfort for the urinary catheter use during the interview. Katz index of independence in activities of daily living (ADL)was measured from 6 items (impairment in bathing, dressing, visiting the toilet, getting up out of a chair, eating, use of incontinence materials) by the observation of researchers [27]. Scores range from 0 to 12, and higher scores indicate more independence in ADL.

Patients with a urinary catheter in place were followed up every day by researchers to evaluate the appropriateness of use. Physicians’ and nurses’ notes were reviewed to clarify indications for the catheters. The total number of inappropriate catheter-days was calculated at discharge as an outcome variable.

### Analysis

Statistical analyses were performed with R Version 3.2.2 [28]. The analysis proceeded in three phases. First, chi-square and independent t tests were used to test the bivariate relationships of demographic factors, present health condition, conditional factors of catheter placement and patients who experienced a change from the appropriate placement to inappropriate use. A *p*-value of < 0.05 was considered statistically significant. Second, a hierarchical multiple regression was then used to identify key related factors associated with changes from appropriate placement to inappropriate use. Finally, to measure the number of inappropriate urinary catheter-days associated with changes from appropriate placement to inappropriate use, a multiple regression analysis was conducted. Variables that were present in *P* < 0.05 on chi-square and independent t tests were entered into the model for adjustment.

Qualitative data of the rationales for urinary catheter placement and the change from appropriate catheter placement to inappropriate use that were collected through medical records or interviews were analyzed to generate categories that represented the different types of rationales for catheter use.

## Results

The mean age of the 156 patients was 78.53±7.25 years, and 59.6% were women. Table 1 shows the characteristics of the patients. Of 156 patients with a catheter placed in the ED, 117 (75%) were classified as appropriate. Compared to patients with inappropriate catheter placement, patients with appropriate placement had fewer depressive symptoms (11.6% vs. 34.4%; p = 0.03) and more dependence in ADL (3.26±3.70 vs. 5.62±4.30; p = 0.003). The most common rationale for appropriate catheter placement was close monitoring of urine output in critically ill patients (Table 2).

**Table 1:** Factors associated with urinary catheter placement and changes from the appropriate to the inappropriate use of a urinary catheter.

Associated factors	Total cohort (n = 156)	Urinary catheter placement			Appropriateness		
		Appropriate(n = 117)	Inappropriate (n = 39)	*P*-value	Not changed (n = 40)	Changed (n = 77)	*P*-value
**Demographic factors**							
Age, mean±sd	78.53±7.25	78.61±7.04	78.31±7.94	0.82	77.78±7.48	78.88±6.87	0.42
Female, n(%)	93(59.6)	74(63.2)	19(48.7)	0.11	27(67.5)	47(61.0)	0.49
**Present health condition**							
Charlson comorbidity index, mean±sd	3.43±2.51	3.42±2.63	3.46±2.15	0.92	3.60±2.89	3.31±2.51	0.57
Cognitive impairment, n(%)	104(68.0)	76(66.1)	28(73.7)	0.38	27(67.5)	49(65.3)	0.81
Depressive symptoms, n(%)	22(17.3)	11(11.6)	11(34.4)	0.03	3(11.1)	8(11.8)	0.92
Katz ADL score on admission, mean±sd	3.85±3.97	3.26±3.70	5.62±4.30	0.003	3.20±72	3.29±3.07	0.92
Urinary tract infection, n(%)	46(29.5)	31(26.5)	15(38.5)	0.15	20(50.0)	11(14.3)	<0.001
Urinary incontinence, n(%)	62(39.7)	51(43.6)	11(28.2)	0.08	20(50.0)	31(40.3)	0.31
**Conditional factors of catheter placement**							
Lack of documentation for rationale of catheter placement, n(%)	86(55.1)	68(58.1)	18(46.2)	0.19	13(32.5)	55(71.4)	<0.001
Caregivers’ perspective of convenience, mean±sd	8.69±2.87	8.67±2.86	8.74±2.93	0.91	7.45±3.60	9.33±2.18	0.005
Patients’ perspective of convenience, mean±sd	8.18±3.36	8.36±3.25	7.61±3.69	0.30	6.54±4.42	9.09±2.32	0.009
Patients’ perspective of comfort, mean±sd	7.72±3.70	7.75±3.78	7.61±3.5	0.85	6.96±4.20	8.09±3.57	0.19

*Katz ADL*, Katz index of independence in activities of daily living

**Table 2:** Rationales for appropriate and inappropriate urinary catheter placement.

Indications of urinary catheter placement	Urinary catheterization, n(%)
**Appropriateness** (n = 117; 75%)	
Close monitoring of urine output in critically ill patients	42(35.8%)
Hip fracture	31(26.5%)
Urinary retention or bladder outlet obstruction	18(15.4%)
Altered mental status or unresponsive	12(10.3%)
Administration of medication into bladder flush during bleeding	6(5.1%)
Preoperative catheter insertion for patients going directly to the operating department	4(3.5%)
Urinary incontinence in the presence of open sacral or perineal wounds	3(3.4%)
**Inappropriateness** (n = 39; 25%)	
Convenience of care	15(38.5%)
Urine specimen collection	9(23.1%)
Prevention of urinary retention	8(20.5%)
No evident reason for urinary catheter use	5(12.8%)
No need for urinary-output monitoring	2(5.1%)

Of 117 patients with appropriate catheter placement, 77 patients (65.8%) experienced a change from appropriate placement to inappropriate use, with a mean duration of 2.88±1.56 days. The common rationales for changing from appropriate urinary catheter placement to inappropriate use were post-operation for hip fracture (36.3%) and no longer needing to monitor urine output (27.2%).

Patients who experienced a change from appropriate placement to inappropriate use had fewer urinary tract infection diagnoses (14.3% vs. 50.0%; *p* <0.001), a lack of a record of the indication for catheter placement (71.4% vs. 32.5%; *p* <0.001), and an increase in patients’ (9.09±2.32 vs. 6.54±4.42; *p =* 0.009) and caregivers’ perspective of convenience (9.33±2.18 vs. 7.45±3.60; *p =* 0.005) compared with patients did not experience a change (Table 1). The hierarchical logistic regression of factors associated with patients who experienced a change from appropriate catheter placement to inappropriate use is summarized in Table 3. In the first model, patients who experienced a change from appropriate placement to inappropriate use were regressed on age and gender. Neither of the demographic factors significantly contributed to the equation and accounted for 0.7% of the variance. In the second model, only urinary tract infection diagnosis (OR = 0.07; 95% CI = 0.02–0.28; *p* <0.001) significantly contributed to the equation, and present health condition added an additional 21.7% of the variance in the change from appropriate catheter placement to inappropriate use. In the third model, urinary tract infection diagnosis (OR = 0.15; 95% CI = 0.03–0.77; *p* = 0.02) and no record of the indication for catheter placement (OR = 4.76; 95% CI = 1.20–18.90; *p* = 0.02) significantly contributed to the equation. Patients’ and caregivers’ perspective of convenience did not reach statistical significance. Conditional factors of catheter placement added an additional 13.5% to the variance, and all variables together explained 35.9% of the variance in the change from appropriate catheter placement to inappropriate use.

**Table 3:** Hierarchical multiple regression of associated factors of changes from the appropriate to the inappropriate use of a urinary catheter.

Associated factors	Model 1 OR (95%CI)	Model 2 OR (95%CI)	Model 3 OR (95%CI)
Step 1: Demographic factors			
Age	1.02 (0.96–1.08)	1.02 (0.94–1.11)	1.04 (0.94–1.14)
Female	0.74 (0.33–1.67)	0.85 (0.26–2.70)	0.69 (0.17–2.81)
**R**^**2**^	**0.7%**		
***F***	**1.15**		
Step 2: Present health condition			
Charlson comorbidity index		0.84 (0.69–1.02)	0.90 (0.72–1.13)
Cognitive impaired		3.26 (0.89–11.85)	2.52 (0.51–12.51)
Depressive symptoms		1.31 (0.24–6.96)	5.49 (0.42–17.39)
Katz ADL score on admission		0.89 (0.78–1.04)	1.06 (0.86–1.28)
Urinary tract infection		0.07 (0.02–0.28)***	0.15 (0.03–0.77)*
Urinary incontinence		0.55 (0.16–1.79)	0.54 (0.12–2.35)
**R**^**2**^		**22.4%**	
**ΔR**^**2**^		**21.7%**	
***F***		25.41**	
Step 3: Conditional factors of catheter placement			
Lack of documentation for rationale of catheter placement			4.76 (1.20–18.90)*
Caregivers’ perspective of convenience			1.09 (0.88–1.34)
Patients’ perspective of convenience			1.23 (0.97–1.56)
Patients’ perspective of comfort			1.13 (0.93–1.37)
**R**^**2**^			**35.9%**
**ΔR**^**2**^			**13.5%**
***F***			38.13***

*Katz ADL*, Katz index of independence in activities of daily living

* p < .05

** p < .01

*** p < .001

Compared to patients who did not experience a change, patients who experienced a change from appropriate placement to inappropriate use had longer inappropriate catheter-days (β = 5.34; 95% CI: 3.72–6.97; *p* <0.001). This difference remained significant in a multivariable analysis adjusting for confounding factors (urinary tract infection diagnosis, presence of the rationale for urinary catheter placement, patients’ and caregivers’ perspective of convenience).

## Discussion

Previous studies described interventions to reduce the inappropriate use of urinary catheters in the ED, and all studies focused on the time of placement. No study has addressed the change from appropriate urinary catheter placement to inappropriate use and associated factors in hospitalized older patients. This study evaluated the dynamic change in catheter use and provides a more complete perspective on the use of catheters. In addition, this study evaluated the change from appropriate catheter placement to inappropriate use and demonstrated a significant association with a prolongation of inappropriate catheter-days.

This study shows that 65.8% of hospitalized older patients experienced a change from appropriate urinary catheter placement to inappropriate use, and the common rationales were “post-operation for hip fracture” and “no longer a need to monitor urine output”. These results are consistent with previous studies that reported that urinary catheters are commonly left in place when they are no longer needed [17, 29]. However, our study emphasizes the concept of transitional care, which is defined as a set of actions designed to ensure the coordination and continuity of health care as patients transfer between different locations or different levels of care within the same location [30]. Healthcare providers should pay attention and re-evaluate the necessity of urinary catheter use when patients undergo surgery or are diagnosed with a critical condition, to disrupt inappropriate catheter use.

The hierarchical logistic regression analysis showed that fewer patients with a diagnosis of urinary tract infection suffered from a change in appropriate urinary catheter placement to inappropriate use. The HICPAC has already begun to announce and educate that “urinary tract infection” is not an indication for the use of urinary catheters, and the risk of urinary catheter use outweighs the benefit [6]. This announcement may have led to fewer patients with a diagnosis of urinary tract infection undergoing a change from appropriate placement to inappropriate use. Of note, a lack of documentation of the rationale for catheter placement was significantly associated with a change from appropriate placement to inappropriate use. It is reasonable to assume that physicians will encounter a dilemma when deciding to remove urinary catheters because they did not know why the patient needed a urinary catheter. Therefore, the physician may prefer not to remove urinary catheters to prevent the inappropriate removal of urinary catheters. More research is needed to clarify physicians’ decision to remove urinary catheters. It would also be interesting to know if the responsible physician was alerted to the presence of the urinary catheter on the ward round. Lack of documentation may indicate that physicians did not pay attention to urinary catheter use. Continuing education in the ED may be needed to enhance physicians’ appropriate attitude related to the use of urinary catheters. In addition, the hospital should have clear regulations for the documentation of the use of urinary catheters.

Our study has a few limitations. First, the nature of secondary analysis limits the availability of comprehensive information, for example, physician’s preference of the culture context of practice for catheter use in the ED cannot be obtained from the original data. Second, during daily follow-ups, contact between the researchers and the patients’ physicians may have served as a potential reminder about appropriate catheter removal, thus underestimating the actual situation. Third, although we did not determine sample size before study, a post hoc power analysis is carried out and the power of this study is 0.88. Finally, the study excluded some patients in whom the urinary catheter was removed between 24 and 48 hours of admission; it is possible that the incidence of changing from appropriate placement to inappropriate use was underestimated.

## Conclusions

In summary, addressing inappropriate urinary catheter use in the ED is an important aspect of patient safety. Substantial opportunities reside in changing from appropriate placement to inappropriate use and reducing further inappropriate catheter-days. We suggest that hospitals not only pay attention to urinary catheter placement in the ED but also assess the dynamic change in urinary catheter use. Catheter reminding intervention included a daily checklist or verbal/written reminder to assess continued catheter need, a sticker reminder on the patient’s chart or an electronic reminder that a catheter is still in place. This study highlights the construction and deployment of catheter reminding intervention to notify clinicians of patients experiencing urinary catheter use. Although, it might be hard for clinicians to assess appropriateness of urinary catheter use every day. This study shows critically important as limiting reassessment appropriateness of urinary catheter use no more than 3 days after placement appear to be the effective approach to prevent inappropriate use. Besides, to guarantee appropriate catheter use, the formulation of a hospital-level clinical policy in the use of urinary catheters is extremely important and should include concrete regulations of documentation and specific protocols for the management of urinary catheters in the ED for this vulnerable population.

## Supporting information

S1 File ER de-identified data set. (XLS)
